# Supplementary material for: Heat adaptation of phage T7 under an extended genetic code
Source: Virus Evol. 2021 Dec 1;7(2):veab100. doi: 10.1093/ve/veab100 (PMC8923235; doi:10.1093/ve/veab100)
Supplement: veab100_Supp [file veab100_supp.zip › Sequencing_Analysis.zip!/Sequencing_Analysis/A3/evidence/JC_SIDE_1_59.html]

BRESEQ :: Evidence


| New junction evidence | | | | | | | | | | | |
| --- | --- | --- | --- | --- | --- | --- | --- | --- | --- | --- | --- |
|  | | seq id | position | reads (cov) | reads (cov) | score | skew | freq | annotation | gene | product |
| \* | ? | NC\_001604.1 | = 7530 | 2179 (1.250) | 29 (0.020) | 24/580 | 31.0 | 1.4% | intergenic (–/–) | *–/–* | –/– |
|  | ? | NC\_001604.1 | 8037 = | 2008 (1.190) |  |  |  |  | intergenic (–/–) | *–/–* | –/– |
| Rejected: Coverage evenness skew score above cutoff. | | | | | | | | | | | |
| Rejected: Frequency below/above cutoff threshold. | | | | | | | | | | | |

Only 100 of 2195 total aligned reads displayed.  

`GGTATGGGGTACAAAAGGTCTGGCTAATGAAGGTAAAGTGATTGGTTTTGAGGTGCTTCTTGAGAGTGGTCGTTTAGTTAACGCCACGAATATCTCTCGCGCCTTAATGGATGAGTTCACTGAGACAGTAAAAGAGGCCACCCTAAGTCAATGGGGATTCTTTAGCCCATACGGTATTGGCGACAACGATGCTTGTACTATTAACCCTTACGATGGCTGGGCGTGTCAAATTAGCTACATGGAGGAAACACCTGATGGCTCTTTGCGGCACCCATCGTTCGTAATGTTCCGTGGCACCGAGGACAACCCTCAAGAGAAAATGTAATCACACTGGCTCACCTTCGGGTGGGCCTTTCTGCGTTTATAAGGAGACACTTTATGTTTAAGAAGGTTGGTAAATTCCTTGCGGCTTTGGCAGCTATCCTGACGCTTGCGTATATTCTTGCGGTATACCCTCAAGTAGCACTAGTAGTAGTTGGCGCTTGTTACTTAGCGGCA‑‑GTGTGTGCTTGCGTGTGGAGTATAGTTAACTGGTAATACGACTCACTAAAGGAGGT‑‑ACACACCATGATGTACTTAATGCCATTACTCATCGTCATTGTAGGAT  >  NC_001604.1/7230‑7830`  

                                                                                                                                                                                                                                                                                                            `|`                                                                                                                                                                                                                                                                                                                  
`ggTATTGGGTACAAAAGGTCTGGCTAATGAAGGTAAAGTGATTGGTTTTGAGGTGCTTCTTGAGAGTGGTCGTTTAGTTAACGCCACGAATATCTCTCGCGCCTTAATGGATGAGTTCACTGAGACAGTAAAAGAGGCCACCCTAAGTCAATGGGGATTCTTTAGCCCATACGGTATTGGCGACAACGATGCTTGTACTATTAACCCTTACGATGGCTGGGCGTGTCAAATTAGCTACATGGAGGAAACACCTGATGGCTCTTTGCGGCACCCATCGTTCGTAATGTTCCGTGGCACCGAg                                                                                                                                                                                                                                                                                                                  >  1:33281/1‑301 (MQ=255)`  
   `aTGGTGTACAAAAGGTCTGGCTAATGAAGGTAAAGTGATTGGTTTTGAGGTGCTTCTTGAGAGTGGTCGTTTAGTTAACGCCACGAATATCTCTCGCGCCTTAATGGATGAGTTCACTGAGACAGTAAAAGAGGCCACACTAAGTCAATGGGGATTCTTTAGCCCATACGGTATTGGCGACAACGATGCTTGTACTATTAACCCTTACGATGGCTGGGCGTGTCAAATTAGCTACATGGAGGAAACACCTGATGGCTCTTTGCGGCACCCATCGTTCGTAATGTTCCGTGGCACCGAGGAc                                                                                                                                                                                                                                                                                                               <  1:61789/301‑1 (MQ=255)`  
               `agacagggCTAATGAATGTAAAGTGATTTGTTTTGAGGTGCTTCTTGAGAGTGGTCGTTTAGTTAACGCCACGAATATCTCTCGCGCCTTAATGGATGAGTTCACTGAGACAGTAAAAGAGGCCACCCTAAGTCAATGGGGATTCTTTAGCCCATATGGTATTGGCGACAACGATGCTTGTACTATTAACCCTTACGATGGCTGGGCGTGTCAAATTAGCTACATGGAGGAAACACCTGATGGCTCTTTGCGGCACCCATCGTTCGTAATGTTCCGTGGCACCGAGGACAACCCTCAagag                                                                                                                                                                                                                                                                                                   <  1:28084/295‑1 (MQ=255)`  
               `aGGTCTGGCTAATGAAGGTAAAGTGATTGGTTTTGAGGTGCTTCTTGAGAGTGGTCGTTTAGTTAACGCCACGAATATCTCTCGCGCCTTAATGGATGAGTTCACTGAGACAGTAAAAGAGGCCACCCTAAGTCAATGGGGATTCTTTAGCCCATACGGTATTGGCGACAACGATGCTTGTACTATTAACCCTTACGATGGCTGGGCGTGTCAAATTAGCTACATGGAGGAAACACCTGATGGCTCTTTGCGGCACCCATCGTTCGTAATGTTCCGTGGCACCGAGGACAACCCTCAagag                                                                                                                                                                                                                                                                                                   <  1:214273/301‑1 (MQ=255)`  
                 `ggcTGGCTAATGAAGGTAAAGGGAGTGGTTTTGAGGTGCTACTTGTGAGTGGTCGTTTAGTAAACGCCCCGAATATCTCTCGCGCCTTAATGGATGAGTTCACTTAGACAGTAAAAGAGGCCACCCTAAGTCAATGGGGATTCTTTATCCCATACGGTATTGGCGACAACGATGCTTGTACTATTAACCCTTACGATGGCTGGGCGTGTCAAATTAGCTACATGGAGGAAACACCTGATGGCTCTTTGCGGCACCCATCGTTCGTTATGTTCCGTGGCACCGAGGACAACACTCAAGAGaa                                                                                                                                                                                                                                                                                                 <  1:256551/299‑1 (MQ=255)`  
                     `ggCTAATGAAGGTAAAGTGATTGGTTTTGAGGTGCTTCTTGAGAGTGGTCGTTTAGTTAACGCCACGAATATCTCTCGCGCCTTAATGGATGAGTTCACTGAGACAGTAAAAGAGGCCACCCTAAGTCAATGGGGATTCTTTAGCCCATACGGTATTGGCGACAACGATGCTTGTACTATTAACCCTTACGATGGCTGGGCGTGTCAAATTAGCTACATGGAGGAAACACCTGATGGCTCTTTGCGGCACACATCGTTCGTAATGTTCCGTGGCACCGAGGACAACCCTCAAGAGAAAATg                                                                                                                                                                                                                                                                                             >  1:128185/1‑301 (MQ=255)`  
                                       `gATTGGTTTTGAGGTGCTTCTTGAGAGTGGTCGTTTAGTTAACGCCACGAATATCTCTCGCGCCTTAATGGATGAGTTCACTGAGACAGTAAAAGAGGCCACCCTAAGTCAATGGGGATTCTTTAGCCCATACGGTATTGGCGACAACGATGCTTGTACTATTAACCCTTACGATGGCTGGGCGTGTCAAATTAGCTACATGGAGGAAACACCTGATTGCTCTTTGCGGCACCCATCGTTCGTAATGTTCCGTGGCACCGAGGACAACCCTCAAGAGAAAATGTAATCACACTGGCTCAcc                                                                                                                                                                                                                                                                           >  1:43626/1‑301 (MQ=255)`  
                                            `ataagagacagCTTCTTGAGAGTGTTCTTTTAGTTAACGCCACTAATATCTCTCGCGCCTTAAAGGATTTGTTCACTGAGACAGTAAAAGAGGCCACCCTAAGTCAATGTGTATTCTTTAGCCCATACGGTTTTGGCTACAACGATGCTTGTACTTTTAACCCTTACGATGGCTGGGCGTGTCAATTTAGCTACATGGAGGAAACACCTGATGGCTCTTTGCGCCACCCCTCGTTCGTAATGTTCCGTGGCACCGAGGACAACCCTCAAGAGAAAATGTAATCACACTGGCTCACATTCgg                                                                                                                                                                                                                                                                      <  1:338786/291‑1 (MQ=255)`  
                                                         `tcttGATAGTGGTCGTTTAGTTAACGCCACGAATATCTCTCGCGCCTTAATGGATGAGTTCACTGAGACAGTAAAAGAGGCCACCCTAAGTCAATGGGGATTCTTTAGCCCATACGGTATTGGCGACAACGATGCTTGTACTATTAACCCTTACGATGGCTGGGCGTGTCAAATTAGCTACATGGAGGAAACACCTGATGGCTCTTTGCGGCACCCATCGTTCGTAATGTTCCGTGGCACCGAGGACAACCCTCAAGAGAAAATGTAATCACACTGGCTCACCTTCGGGTGGGCCTGTCtc                                                                                                                                                                                                                                                         >  1:267841/1‑300 (MQ=255)`  
                                                               `gagTGGTCGTTTAGTTAACGCCACGAATATCTCTCGCGCCTTAATGGATGAGTTCACTGAGACAGTAAAAGAGGCCACCCTAAGTCAATGGGGATTCTTTAGCCCATACGGTATTGGCGACAACGATGCTTGTACTATAAACCCTTACGATGGCTGGGCGTGTTAAATTAGCTACATGGAGGAAACACCTGATGGCTCTTTGCGGCACCCATCGTTCGTAATGTTCCGTGGCACCGAGGACAACCCTCAAGAGAAAATGTAATCACACTGGCTCACCTTCGGGTGGGCCTTTCTGAGTTta                                                                                                                                                                                                                                                   >  1:124112/1‑301 (MQ=255)`  
                                                                 `gTGGTCGTTTAGTTAACGCCACGAATATCTCTCGCGCCTTAATGGATGAGTTCACTGAGACAGTAAAAGAGGCCACCCTAAGTCAATGGGGATTCTTTAGCCCATACGGTATTGGCGACAACGATGCTTGTACTATTAACCCTTACGATGGCTGGGCGTGTCAAATTAGCTACATGGAGGAAACACCTGATGGCTCTTTGCTGCACCCATCGTTCGTAATGTTCCGTGGCACCGAGTACAACCCTCAAGAGAAAATGTAATCACACTGGCTCACCTTCTGGTGGGCCTTTCTGCGTTtata                                                                                                                                                                                                                                                 >  1:202995/1‑301 (MQ=255)`  
                                                                                       `gactctctctcGCGCCTTAATGGATGAGTTCACTGAGAAAGTAAAAGAGGCCACCCTAAGTCAATGGGTATTCTTTAGCCCATACGGTATTGGCGACAACGATGCTTGTACTATTAACCCTTACGAGGGCTGGGCGTGTCAAATTAGCTACATGGAGGAAACACCTGATGGCCCTTTGCGGCACCCATCGTTCGTAATGTTCCGTGGCACCGAGGACAACCATCAAGAGAAAATGTAATCACACTGGCTCACATTCGGGTGGGCCTTTCTGCGTTTATAAGGAGACACTTTATGTTTaaga                                                                                                                                                                                                                           <  1:290839/296‑1 (MQ=255)`  
                                                                                       `aaatatcctccgcgCCTTAATGGATGAGTTCCCTGAGACAGTAAAAGAGGCCACCCTAAGTCAAGGGGGATTCTTTAGACCATACGGTATTGGCGACAACGATGCTTGTACTATTAACCCTTACGATGGCTGGGCGTGTCAAATTAGCTACATGGAGGAAACACCTGATGGCTCTTTGCGGCACCAATCGTTCGTAATGTTCCGTGGCACCGAGGACAACCCTCAAGAGAAAATGTAATCACACTGGCTCACCTTCGGGTGGGCCTTTCTGCGTTTATAAGGAGACACTTTATGTTTaaga                                                                                                                                                                                                                           <  1:205966/291‑1 (MQ=255)`  
                                                                                                 `cgcgCCTTAATGGATGAGTTCACTGAGACAGTAAAAGAGGCCACCCTAAGTCAATGGGTATTCTTTATCCCATACGGTATTGGCGACAACGATGCTTGTACTATTAACCCTTACGATGGCTGGGCGTGTCAAATTAGCTTCATGGAGGAAACACCTGATGGCTCTTTGCGGCACCCATCGTTCGTAATGTTCCGTGGCACCGAGGACAACCCTCAAGAGAAAATGTAATCACACTGGCTCACCTTCGGGTGGGCCTTTCTGCGTTTATAAGGAGACACTTTATGTTTAAGAAGGTTGGTaa                                                                                                                                                                                                                 <  1:322338/301‑1 (MQ=255)`  
                                                                                                 `cgcgCCTTAATGGATGAGTTCACTGAGACAGTAAAAGAGGCCACCCTAAGTCAATGGGGATTCTTTAGCCCATACGGTATTGGCGACAACGATGCTTGTACTATTAACCCTTACGATGGATGGGCGTGTCAAATTAGCTACATGGAGGAAACACCTGATGGCTCTTTGCGGCACCCATCGTTCGTAATGTTCCGTGGCACCGAGGACAACCATCAAGAGAAAATGTAATCACACTGGCTCACCTTCGGGTGGGCCTTTCTGCGTTTATAAGGAGACACTTTATGTTTAAGAAGGTTGGTaa                                                                                                                                                                                                                 <  1:176279/301‑1 (MQ=255)`  
                                                                                                     `ccTTAATGGATGAGTTCACTGAGACAGTAAAAGAGGCCACCCTAAGTCAATGGTGATTCTTTAGCCCATACGGTATTGGCGACAACGATGCTTGTACTATTAACCCTTACGATTGCTGGGCGTGTCAAATTAGCTACATGGAGGAAACACCTGATGGCTCTTTGCGGCACCCATCGTTCGTAATGTTCCGTGGCACCGAGGACAACCCTCAAGAGAAAATGTAATCACACTGGCTCACCTTCGGGTGGGCCTTTCTGCGTTTATAAGGAGACACTTTATGTTTAAGAAGGTTGGTAAATTc                                                                                                                                                                                                             >  1:119451/1‑301 (MQ=255)`  
                                                                                                           `tGGATGAGTTCACTGAGACAGTAAAAGAGGCCACCCTAAGTCAATGGGGATTCTTTAGCCCATACGGTATTGGCGACAACGATGCTTGTACTATTAACCCTTACGATGGCTGGGCGTGTCAAATTAGCTACATGGAGGAAACACCTGATGGCTCTTTGCGGCACCCATCGTTCGTAATGTTCCGTGGCACCGAGGACAACCCTCAAGAGAAAATGTAATCACACTGGCTCACCTTCGGGTGGGCCTTTCTGCGTTTATAAGGAGACACTTTATGTTTAAGAAGGTTGGTAAATTCCTTGCg                                                                                                                                                                                                       >  1:113480/1‑301 (MQ=255)`  
                                                                                                            `ggATGAGTTCACTGAGACAGTAAAAGAGGCCACCCTAAGTCAATGGGGATTCTTTAGCCCATACGGTATTGGCGACAACGATGCTTGTACTATTAACCCTTACGATGGCTGGGCGTGTCAAATTAGCTACATGGAGGAAACACCTGATGGCTCTTAGCGTCACCCATCGTTCGTAATGTTCCGTGGCACCGAGGACAACCCCCAAGAGAAAATGTAATCACACTGGCTCAACTTCGGGTGGGCCTTTCTGCGTTTATAAGGAGAAAGTTTATGGTTAAGAAGGTGGGTAAATTctgttcgg                                                                                                                                                                                                      >  1:17973/1‑294 (MQ=255)`  
                                                                                                                           `gaCAGTAAAAGAGGCCACCCTAAGTCAATGGGGATTCTTTAGCCCATACGGTATTGGCGACAACGATGCTTGTACTATTAACCCTTACGATGGCTGGGCGTGTCAAATTAGCTACATGGAGGAAACACCTGATGGCTCTTTGCGGCACCCATCGTTCGTAATGTTCCGTGGCACCGAGGACAACCCTCAAGAGAAAATGTAATCACACTGGCTCACCTTCGGGTGGGCCTTTCTGCGTTTATAAGGAGACACTTTATGTTTAAGAAGGTTGGTAAATTCCTTGCGGCTTTGGCAGCTATcc                                                                                                                                                                                       <  1:276338/301‑1 (MQ=255)`  
                                                                                                                               `gTAAAAGAGGCCACCCTAAGTCAATGGGGATTCTTTAGCCCATACGGTATTGGCGACAACGATGCTTGTACTATTAACCCTTACGATGGCTGGGCGTGTCAAATTAGCTACATGGAGGAAACACCTGATGGCTCTTTGCGGCACCCATCGTTCGTAATGTTCCGTGGCACCGAGGACAACCCTCAAGAGAAAATGTAATCACACTGGCTCACCTTCGGGTGGGCCTTTCTGCGTTTATAAGGAGACACTTTATGTTTAAGAAGGTTGGTAAATTCCTTGCGGCTTTGGCAGCTATCCTGAc                                                                                                                                                                                   >  1:84210/1‑301 (MQ=255)`  
                                                                                                                               `gTAAAAGAGGCCACCCTAAGTCAATGGGGATTCTTTAGCCCATACGGTATTGGCGACAACGATGCTTGTACTATTAACCCTTACGATGGCTGGGCGTGTCAAATTAGCTACATGGAGGAAACACCTGATGGCTCTTTGCGGCACCCATCGTTCGTAATGTTCCGTGGCACCGAGGACAACCCTCAAGAGAAAATGTAATCACACTGGCTCACCTTCCGGTGGGCCTTTCTGCGTTTATAAGGAGACACTTTATGTTTAAGAAGGTTGGTAAATTCCTTGCGGCTTTGGCAGCTATCCTGAc                                                                                                                                                                                   >  1:235748/1‑301 (MQ=255)`  
                                                                                                                                     `gagGCCACCCTAAGTCAATGGGGATTCTTTAGCCCATACGGTATTGGCGACAACGATGCTTGTACTATTAACCCTTACGATGGCTGGGCGTGTCAAATTAGCTACATGGAGGAAACACCTGATGGCTCTTTGCGGCACCCATCGTTCGTAATGTTCCGTGGCACCGAGGACAACCCTCAAGAGAAAATGTAATCACACTGGCTCACCTTCGGGTGGGCCTTTCTGCGTTTATAAGGAGACACTTTATGTTTAAGAAGGTTGGTAAATTCCTTGCGGCTTTGGCAGCTATACTGActgtctc                                                                                                                                                                             >  1:211983/1‑295 (MQ=255)`  
                                                                                                                                           `aCCCTAAGTCAATGGGGATTCTTTAGCCCATACGGTATTGGCGACAACGATGCTTGTACTATTAACCCTTACGATGGCTGGGCGTGTCAAATTAGCTACATGGAGGAAACACCTGATGGCTCTTTGCGGCACCCATCGTTCGTAATGTTCCGTGGCACCGAGGACAACCCTCAAGAGAAAATGTAATCACACTGGCTCACCTTCGGGTGGGCCTTTCTGCGTTTATAAGGAGACACTTTATGTTTAAGAAGGTTGGTAAATTCCTTGCGGCTTTGGCAGCTATCCTGACGCTTGCGTatat                                                                                                                                                                       >  1:209178/1‑301 (MQ=255)`  
                                                                                                                                            `cccTAAGTCAATGGGGATTCTTTAGCCCATACGGTATTGGCGACAACGATGCTTGTACTATTAACCCTTACGATGGCTGGGCGTGTCAAATTAGCTACATGGAGGAAACACCTGATGGCTCTTTGCGGCACCCATCGTTCGTAATGTTCCGTGGCACCGAGGACAACCCTCAAGAGAAAATGTAATCACACTGGCTCACCTTCGGGTGGGCCTTTCTGCGTTGATAAGGAGACACTTTATGTTTAAGAAGGTTGGTAAATTCCTTGCGGCTTTGGCAGCTATCCTGACGCTTGCGGAGAtt                                                                                                                                                                      >  1:244747/1‑301 (MQ=255)`  
                                                                                                                                                `aaGTCAATGGGGATTCTTTAGCCCATACGGTATTGGCGACAAGAATGGTTGGACTATTAACCCTTACGATGGCTGGGCGTGTCAAATTAGCAACATGGAGGAAACACCTGATGGCTCTTAGCGGCACCCATCGTTCGTAATGTGCCGTGGCACCGAGGACAACCCACAAGAGAAAATGTAATCACACTGGCTCACCGTCGGGTGGGCATTTCTGTGTTTATAAGGAGACACTTTATGTTTAAGAAGGTTGGTAAATTCCTTGCGGCTTTGGCAGCTATCCTGACGCTTGCGTATATTCTTg                                                                                                                                                                  <  1:103143/301‑1 (MQ=255)`  
                                                                                                                                                   `tCAATGGGGATTCTTTAGCCCATACGGTATTGGCGACAACGATGCTTGTACTATTAACCCTTACGATGGCTGGGCGTGTCAAATTAGCTACATGGAGGAAACACCTGATGGCTCTTTGCGGCACCCATCGTTCGTAATGTTCCGTGGCACCGAGGACAACCCTCAAGAGAAAATGTAATCACACTGGCTCACCTTCGGGTGGGCCTTTCTGCGTTTATAAGGAGACACTTTATGTTTAAGAAGGTTGGTAAATTCCTTGCGGCTTTGGCAGCTATCCTGACGCTTGCGTATATTCTTGCgg                                                                                                                                                               <  1:146627/301‑1 (MQ=255)`  
                                                                                                                                                          `ggAATCTTTAGCCCCTACGGTATTTGCGACAACTATGCTTGTACTATTCTCCATTACTATGGCTCGGCTTGACAAATTAGCTACATGTATGTAACACCTGATGTCTCTTTCCGGCACCCATCTTTCGTAATGTTCCGTGGCACCTAGGACAACCCTCAAGAGTACATGTAAGCACACTGTCTCACCTGCGGGTCGGCCTTTCTGCGTTTATAAGTACACACTTTATGTTTAATAAGGTTGGTAAATTCCTTGCGGCTTTGGCAGCTATCCTGACGCTTGCGTATATTCTTGCGGTATAccc                                                                                                                                                        <  1:224798/301‑1 (MQ=255)`  
                                                                                                                                                          `acagtCTTTAGCCCATACGGTATTGGCGACAACGATGCTTGTACTATTAACCCTTACGATGGCTGGGCGTGTCAAATTAGCTACATGGAGGAAACACCTGATGGCTCTTTGCGGCACCCATCGTTCGTAATGTTCCGTGGCACCGAGGACAACCCTCAAGAGAAAATGTAATCACACTGGCTCACCTTCGGGTGGGCCTTTCTGCGTTTATAAGGAGACACTTTATGTTTAAGAAGGTTGGTAAATTCCTTGCGGCTTTGGCAGCTATCCTGACGCTTGCGTATATTCTTGCGGTATAccc                                                                                                                                                        <  1:248002/297‑1 (MQ=255)`  
                                                                                                                                                           `gATTCTTTAGCCCATACGGTATTGGCGACAACGATGCTTGTACTATTAACCCTTACGATGGCTGGGCGTGTCAAATTAGCTACATGGAGGAAACACCTGATGGCTCTTTGCGGCACCCATCGTTCGTAATGTTCCGTGGCACCGAGGACAACCCTCAAGAGAAAATGTAATCACACTGGCTCACCTTCTGGTGGGCCTTTCTGCGTTTATAAGGATACACTTTATGTTTAAGAAGGTTGGTAAATTCCTTGCGGCTTTGGCAGCTATCCTGACGCTTGCGTATATTCTTTCGGTATACCCt                                                                                                                                                       <  1:191591/301‑1 (MQ=255)`  
                                                                                                                                                           `aagagacaggCCCATACGGTATTGGCGACAACGATGCTTGTACTATTAACCCTTACGATGGCTGGGCGTGTCAAATTAGCTACATGGAGGAAACACCTGATGGCTCTTTGCGGCACCCATCGTTCGTAATGTTCCGTGGCACTGAGGACAACCCTCAAGAGAAAATGTAATCACACTGGCTCACCTTCGGGTGGGCCTTTCTGCGTTTATAAGGAGACACTTTATGTTTAAGAAGGTTGGTAAATTCCTTGCGGCTTTGGCAGCTATCCTGACGCTTGCGTATATTCTTGCGGTATACCCt                                                                                                                                                       <  1:24149/292‑1 (MQ=255)`  
                                                                                                                                                           `aagagacaggCCCATACGGTATTGGCGACAACGATGCTTGTACTATTAACCCTTACGATGGCTGGGCGTGTCAAATTAGCTACATGGAGGAAACACCTGATGGCTCTTTGCGGCACCCATCGTTCGTAATGTTACGTGGCACCGAGGACAACCCTCAAGAGAAAATGTAATCACACTGGCTCACCTTCGGGTGGGCCTTTCTGCGTTTATAAGGAGACACTTTATGTTTAAGAAGGTTGGTAAATTCCTTGCGGCTTTGGCAGCTATCCTGACGCTTGCGTATATTCTTGCGGTATACCCt                                                                                                                                                       <  1:194221/292‑1 (MQ=255)`  
                                                                                                                                                             `ttCTTTAGCCCATACGGTATTGGCGACAACGATGCTTGTACTATTAACCCTTACGATGGCTTGGCGTGTCAAATTAGCTACATGTAGGAAACACCTGATGGCTCTTTGCGGCACCCATCGTTCGTAATGTTCCGTGGCACCGAGGACAACCCTCAAGAGAAAATGTAATCACACTGGCTCACCTTCGGGTGGGCCTTTCTGCGTTTATAAGGAGACACTTTATGTTTAAGAAGGTTGGTAAATTCCTTGCGGCTTTGGCAGCTATCCTGACGCTTGCGTATATTCTTGCGGTATACCCTCa                                                                                                                                                     >  1:106733/1‑301 (MQ=255)`  
                                                                                                                                                              `tCTTTAGCCCATACGGTATTGGCGACAACGATGCTTGTACTATTAACCCTTACGATGGCTGGGCGTGTCAAATTAGCTACATGGAGGAAACACCTGATGGCTCTTTGCGGCACCCATCGTTCGTAATGTTCCGTGGCACCGAGGACAACCCTCAAGAGAAAATGTAATCACACTGGCTCACCTTCGGGTGGGCCTTTCTGCGTTTATAAGGAGACACTTTATGTTTAAGAAGGTTGGTAAATTCCTTGCGGCTTTGGCAGCTATCCTGACGCTTGCGTATATTCTTGCGGTATACCCTCaa                                                                                                                                                    >  1:258583/1‑301 (MQ=255)`  
                                                                                                                                                              `tCTTTAGCCCATACGGTATTGGCGACAACGATGCTTGTACTATTAACCCTTACGATGGCTGGGCGTGTCAAATTAGCTACATGGAGGAAACACCTGATGGCTCTTTGCGGCACCCATCGTTCGTAATGTTCCGTGGCACCGAGGACAACCCTCAAGAGAAAATGTAATCACACTGGCTCACCTTCGGGTGGGCCTTTCTGCGTTTATAAGGAGACACTTTATGTTTAAGAAGGTTGGTAAATTCCTTGCGGCTTTGGCAGCTATCCTGACGCTTGCGTATATTCTTGCGGTATACCCTCaa                                                                                                                                                    >  1:149769/1‑301 (MQ=255)`  
                                                                                                                                                                `tttAGCCCATACGGTATTGGCGACAACGATGCTTGTACTATTAACCCTTACGATGGCTGGGCGTGTCAAATTAGCTACATGGAGGAAACACCTGATGGCTCTTTGCGGCACCCATCGTTCGTAATGTTCCGTGGCACCGAGGACAACCCTCAAGAGAAAATGTAATCACACTGGCTCACCTTCGGGTGGGCCTTTCTGCGTTTATAAGGAGACACTTTATGTTTAAGAAGGTTGGTAAATTCCTTGCGGCTTTGGCAGCTATCCTGACGCTTGCGTATATTCTTGCGGTATACCCTCAAGt                                                                                                                                                  <  1:47418/301‑1 (MQ=255)`  
                                                                                                                                                                    `gCCCATACGGTATTGGCGACAACGATGCTTGTACTATTAACCCTTACGATGGCTGGGCGTGTCAAATTAGCTACATGGAGGAAACACCTGATGGCTCTTTGCGGCACCCATCGTTCGTAATGTTCCGTGGCACCGAGGACAACCCTCAAGAGAAAATGTAATCACACTGGCTCACCTTCGGGTGGGCCTTTCTGCGTTTATAAGGAGACACTTTATGTTTAAGAAGGTTGGTAAATTCCTTGCGGCTTTGGCAGCTATCCTGACGCTTGCGTATATTCTTGCGGTATACCCTCAAGTAGCa                                                                                                                                              >  1:167214/1‑301 (MQ=255)`  
                                                                                                                                                                     `cccATACGGTATTGGCGACAACGATGCTTGTACTATTAACCCTTACGATGGCTGGGCGTGTCAAATTAGCTACATGGAGGAAACACCTGATGGCTCTTTGCGGCACCCATCGTTCGTAATGTTCCGTGGCACCGAGGACAACCCTCAAGAGAAAATGTAATCACACTGGCTCACCTTCGGGTGGGCCTTTCTGCGTTTATAAGGAGACACTTTATGTTTAAGAAGGTTGGTAAATTCCTTGCGGCTTTGGCAGCTATCCTGACGCTTGCGTATATTCTTGCGGTATACCCTCAAGTAGCAc                                                                                                                                             <  1:153248/301‑1 (MQ=255)`  
                                                                                                                                                                        `aTACGGTATTGGCGACAACGATGCTTGTACTATTAACCCTTACGATGGCTGGGCGTGTCAAATTAGCTACATGGAGGAAACACCTTATGTCTCTTTGCTGCACCCATCGTTCGTAATGTTCCGTGGCACCGAGGACAACCCTCAAGAGAAAATGTAATCACACTGGCTCACCTTCGTGTGGGCCTTTCTGCGTTTATAAGGAGACACTTTATGTTTAATAAGGTTGTTAAATTCCTTTCGGCTTTTGCAGCTATCCTGACGCTTGCGTATCTTCTTGCGGTTTCCCCTCAAGTAGCACtag                                                                                                                                          >  1:336188/1‑301 (MQ=255)`  
                                                                                                                                                                          `aCGGTATTGGCGACAACGATGCTTGTACTATTAACCATTACGATGGCTGGGCGTGTCAAATTAGCTACATGGAGGAAACACCTGATGGCTCTTTGCGGCACCCATCGTTCGTAATGTTCCGTGGCACCGAGGACAACCCTCAAGAGAAAATGTAATCACACTGGCTCACCTTCGGGTGGGCCTTTCTGCGTTTATAAGGAGACACTTTATGTTTAAGAAGGTTGGTAAATTCCTTGCGGCTTTGGCAGCTATCCTGACGCTTGCGTATATTCTTGCGGTATACCCTCAAGTAGCACtgtct                                                                                                                                        >  1:314087/1‑297 (MQ=255)`  
                                                                                                                                                                           `cGGTATTGGCGACAACGATGCTTGTACTATTAACCCTTACGATGGCTTGGCGTGTCAAATTAGCTACATGGATGAATCACCTGATGGCTCTTTGCGGCACCCATCGTTCGTAATGTTCCGTTGCACCGAGGACAA‑CCTCAAGAGAAAATGTAATCACACTGGCTCACCTTCGGGTGGGCCTTTCTTCGTTTATAAGGAGACACTTTATGTTTAAGAATGTTGGTAAATTCCTTGCGGCTTTGGCAGCTATCCTGACGCTTGCGTATATTCTTGCGGTATACCCTCCAGTCGCACtagtagt                                                                                                                                      >  1:68455/1‑301 (MQ=255)`  
                                                                                                                                                                             `gTATTGGCGACAACGATGCTTGTACTATTAACCCTTACGATGGCTGGGCGTGTCAAATTAGCTACATGGAGGAAACACCTGATGGCTCTTTGCGGCACCCATCGTTCGTAATGTTCCGTGGCACCGAGGACAACCCTCAAGAGAAAATGTAATCACACTGGCTCACCTTCGGGTGGGCCTTTCTGCGTTTATAAGGAGACACTTTATGTTTAAGCATGTTGGTAAATTCCTTGCGGCTTTGGCATCTATCCTGACGCTTGCGTATATTCTTTCGGTATACCCTCCAGTATCTCtattagta                                                                                                                                     >  1:283411/1‑301 (MQ=255)`  
                                                                                                                                                                             `gTATTGGCGACAACGATGCTTGTACTATTAACCCTTACGATGGCTGGGCGTGTCAAATTAGCTACATGGAGGAAACACCTGATGGCTCTTTGCGGCACCCATCGTTCGTAATGTTCCGTGGCACCGAGGACAACCCTCAAGAGAAAATGTAATCACACTGGCTCACCTTCGGGTGGGCCTTTCTGCGTTTATAAGGAGACACTTTATGTTTAAGACGGTTGGTAAATTCCTTGCGGCTTTGGCAGCTATCATGACGCTTGCGTATATTCTTGCGGTATACCCTCAAGTAGCACtagtagtt                                                                                                                                     >  1:187928/1‑300 (MQ=255)`  
                                                                                                                                                                             `aaagagacagcAACGATGCTTGTACTATTAACCCTTACGATGGCTGGGCGTGTCAAATTAGCTACATGGAGGAAACACCTGATGGCTCTTTGCGGCACCCATCGTTCGTAATGTTCCGTGGCACCGAGGACAACCCTCAAGAGAAAATGTAATCACACTGGCTCACCTTCGGGTGGGCCTTTCTGCGTTTATAAGGAGACACTTTATGTTTAAGAAGGTTGGTAAATTCCTTGCGGC‑TTGGCAGCTATCCTGACGCTTGCGTATATTCTTGCGGTATACCATCAAGTAGCACtagtagtag                                                                                                                                    <  1:304601/291‑1 (MQ=255)`  
                                                                                                                                                                                `ttGGCGACAACGATGCTTGTACTATTAACCCTTACGAGGGCTGGGCGTGTCAAATTAGCTACATGGAGGAAACACCTGATTGCACTTTGCGGCACCCATCGTTCGTAATGTTCCGTGGCACCGAGGACAACCCTCAAGAGAAAATGTAATCACACTGGCTCACCTTCGGGTTGGCCTTTCTGCGTTTATAAGGAGACACTTTATGTTTAAGAAGGTTGGTAAATTCCTTGCGGCTTTGGCAGCTATCCTGACGCTTGCGTATATTCTTGCGGTATACCCTCAAGTAGCACTAGTAGTAGtt                                                                                                                                  <  1:90063/301‑1 (MQ=255)`  
                                                                                                                                                                                `ttGGCGACAACGATGCTTGTACTATGAACCCTTACGATGGCTGGGCGTGTCAAATTAGCTACATGGAGGAAAAACCTGATGGCTCTTTGCGGCACCCATCGTTCGTAATGTTCCGTGGCACCGAGGACAACCCTCAAGAGAAAATGTAATCACACTGGCTCACCTTCGGGTGGGCCTTTCTGCGTTTATAAGGAGACACTTTATGTTTAAGAAGGTTGGTAAATTCCTTGCGGCTTTGGCAGCTATCCTGACGCTTGCGTATATTCTTGCGGTATACCCTCAAGTAGCACTAGTAGTAGtt                                                                                                                                  <  1:1166/301‑1 (MQ=255)`  
                                                                                                                                                                                  `ggggACAACGATGCTTGTAATTTTTACCCTTACGGATGCTGGGCGTGTCAAATTAGCTACATGGAGGAAACACCTGATGGCTCTTTGCGGCAACCATCGTTCGTAATGTTCCGTGGCAACGAAGACAACCCTCAAGAGAAAATGTAATCACACTGGCTCAACTTCGGGTGGGCCTTTCTGCGTTTATAATGAGACACTTTATGTTTAAGAAGGTTGGTAAATTCCTTGCGGCTTTGGCAGCTATCCTGACGCTTGCGTATATTCTTGCGGTATACCCTCAAGTAGCACTAGTAGTAGTTgg                                                                                                                                <  1:5485/298‑1 (MQ=255)`  
                                                                                                                                                                                  `ggCGACAACGATGCTTGTACTATTAACCCTTACGATGGCTGGGCGTGTCAAATTAGCTACATGGAGGAAACACCTGATGGCTCTTTGCGGCACCCATCGTTCGTAATGTTCCGTGGCACCGAGGACAACCCTCAAGAGAAAATGTAATCACACTGGCTCACCTTCGGGTGGGCCTTTCTGCGTTTATAAGGAGACACTTTATGTTTAAGAAGGTTGGTAAATTCCTTGCGGCTTTGGCAGCTATCCTGACGCTTGCGTATATTCTTGCGGTATACCCTCAAGTAGCACTAGTAGTAGTTgg                                                                                                                                <  1:100049/301‑1 (MQ=255)`  
                                                                                                                                                                                       `cAACGATGCTTGTACTATTAACCCTTACGATGGCTGGGCGTGTCAAATTAGCTACATGGAGGAAACACCTGATGGCTCTTTGCGGCACCCATCGTTCGTAATGTTCCGTGGCACCGAGGACAACCCTCAAGAGAAAATGTAATCACACTGGCTCACCTTCGGGTGGGCCTTTCTGCGTTTATAAGGAGACACTTTATGTTTAAGAAGGTTGGTAAATTCCTTGCGGCTTTGGCAGCTATCCTGACGCTTGCGTATATTCTTGCGGTATACCCTCAAGTAGCACTAGTAGTAGTTGGCGCtt                                                                                                                           >  1:77165/1‑301 (MQ=255)`  
                                                                                                                                                                                             `tGCTTGTACTATTAACCCTTACGATGGCTGGGCGTGTCAAATTAGCTACATGGAGGAAACACCTGATGGCTCTTTGCGGCACCCATCGTTCGTAATGTTCCGTGTCACCGAGGACAACCCTCCAGAGAAAATGTAATCACACTGGCTCACCTTCGGGTGGGCCTTTCTGCGTTTATAAGGAGACACTTTATGTTTAAGAAGGTTGGTAAATTCCTTGCGGCTTTGGCAGCTATCCTGACTCTTGCGTATATTCTTGCGGTATCCCCTCAAGTAGCACTAGTAGTAGTTGGCGCTTGTTACt                                                                                                                     >  1:136351/1‑301 (MQ=255)`  
                                                                                                                                                                                              `gCTTGTACTATTAACCCTTACGATGGCTGGGCGTGTCAAATTAGCTACATGGAGGAAACACCTGATGGCTCTTTGCGGCACCCATCGTTCGTAATGTTCAGTGGCACCGAGGACAACCCTCAAGAGAAAATGTAATCACACTGGCTCACCTTCGGGTGGGCCTTTCTGCGTTTATAAGGAGACACTTTATGTTTAAGAAGGTTGGTAAATTCCTTGCGGCTTTGGCAGCTATCCTGACGCTTGCGTCTATTCTTGCGGTATACCCTCAAGTAGCACTAGTAGTAGTTGGCGCTTGTTACtt                                                                                                                    >  1:26161/1‑301 (MQ=255)`  
                                                                                                                                                                                                `ttGTACTATTAACCCTTACGATGGCTGGGCGTGTCAAATTAGCTACATGGAGGAAACACCTGATGGCTCTTTGCGGCACCCATCGTTCGTAATGTTCCGTGGCACCGAGGACAACCCTCAAGAGAAAATGTAATCACACTGGCTCACCTTCGGGTGGGCCTTTCTGCGTTTATAAGGAGACACTTTATGTTTAAGAAGGTTGGTAAATTCCTTGCGGATTTGGCAGCTATCCTGACGCTTGCGTATATTCTTGCGGTATACCCTCAAGTAGCACTAGTAGTAGTTGGCGCTTGTTACTTAg                                                                                                                  <  1:238923/301‑1 (MQ=255)`  
                                                                                                                                                                                                `ttGTACTATTAACCCTTACGATGGCTGGGCGTGTCAAATTAGCTACATGGAGGAAACACCTGATGGCTCTTTGCGGCACCCATCGTTCGTAATGTTCCGTGGCACCGAGGACAACACTCAAGAGAAAATGTAATCACACTGGCTCACCTTCGGGTGGGCCTTTCTGCGTTTATAAGGAGACACTTTATGTTTAAGAAGGTTGGTAAATTCCTTGCGGCTTTGGCAGCTATCCTGACGCTTGCATATATTCTTGCGGTATACCCTCAAGTAGCACTAGTAGTAGTTGGCGCTTGTTACTTAg                                                                                                                  >  1:297575/1‑301 (MQ=255)`  
                                                                                                                                                                                                    `aCTATTAACCCGTACGATGGCTGGTCGTGTCAAAGTAGCTACATGGAGGAAACACCTGATGGCTCTTTGCGGCACCCATCATTCGTAATGTTCATTGGCACCGAGGACAACTCTCAAGAGAAAATGTAATCACACTGGCTCACCTTCGGGTGGGCCTGTCTGCGTGTATAAGGAGACACTTTATGTTTAAGAAGGTTGGTAAATGCCTTGCGGCTTTGGCAGTGATTCTGACGCTTGCGTATATTCTTGCGGTATACCCTCAAGTAGCACTAGTAGTAGTTGGCGCTTGTTACTTAGCGGc                                                                                                              <  1:341631/301‑1 (MQ=255)`  
                                                                                                                                                                                                     `cTATTAACCCTTACGATTGCTGGGCGTGTCAAATTAGCTACATGGAGGAAACACCTGATGGCTCTTTGCGGCACCCATCGTTCGTAATGTTCCGTGGCACCGAGGACAACCCTCAAGAGAAAATGTAATCACACTGGCTCACCTTCGGGTGGGCCTTTCTGCGTTTATAAGGAGACACTTTATGTTTAAGAAGGTTGGTAAATTCCTTGCGGCTTTGGCAGCTATCCTGACTCTTGCGTATATTCTTGCGGTATACCCTCAAGTAGCACtagtctgtctcttatacacatctccgagccca                                                                                                             >  1:308514/1‑273 (MQ=255)`  
                                                                                                                                                                                                        `ttAACCCTTACGATGGCTGGGCGTGTCAAATTAGCTACATGGAGGAAACACCTGATGGCTCTTTGCTGCACCCATCGTTCGTAATGTTCCGTGGCACCGAGGACAACCCTCAAGAGAAAATGTAATCACACTGGCTCACCTTCGGGTGGGCCTTTCTGCGTTTATAAGGAGACACTTTATGTTTAAGAAGGTTGGTAAATTCCTTGCGGCTTTGGCAGCTATCCTGACGCTTGCGTATATTCTTGCGGTATACCCTCAAGTAGCACTAGTAGTAGTTGGCGCTTGTTACTTAGCGGCA‑‑gtg                                                                                                        <  1:316766/301‑1 (MQ=255)`  
                                                                                                                                                                                                        `ttAACCCTTACGATGGCTGGGCGTGTCAAATTAGCTACATAGAGGAAACACCTGATGGCTCTTTGCGGCACCCATCGTTCGTAATGTTCCGTGGCACCGAGGACAACCCTCAAGAGAAAATGTAATCACACTGGCTCACCTTCGGGTGGGCCTTTCTGCGTTTATAAGGAGACACTTTATGTTTAAGAAGGTTGGTAAATTCCTTGCGGCTTTGGCAGCTATCCTGACGCTTGCGTATATTCTTGCGGTATACCCTCAAGTAGCACTAGTAGTAGTTGGCGCTTGTTACTTAGCGGCA‑‑gtg                                                                                                        <  1:5247/301‑1 (MQ=255)`  
                                                                                                                                                                                                              `cTTACGATGGCTGGGCGTGTCAAATTAGCTACATGGAGGAAATACCTGATGGCTCTTTGCGGCACCCATCGTTCGTAATGTTCCGTGGCACCGAGGACAACCCTCAAGAGAAAATGTAATCACACTGGCTCACCTTCGGGTGGGCCTTTCTGCGTTTATAAGGAGACACTTTATGTTTAAGAAGGTTGGTAAATTCCTTGCGGCTTTGGCAGCTATCCTGACGCTTGCGTATATTCTTGCGGTATACCCTCAAGTAGCACTAGTAGTAGTTGGCGCTTGTTACTTAGCGGCA‑‑GTGTGTGCt                                                                                                  <  1:133439/301‑1 (MQ=255)`  
                                                                                                                                                                                                              `cTTACGATGGCTGGGCGTGTCAAATTAGCTACATGGAGGAAACACCTTATGGCTCTTTGCGGCACCCATCGTTCGTAATGTTCCGTGGCACCGAGGATAACCCTCAAGAGAAAATGTAATCACACTGGCTCACCTTCGGGTGGGCCTTTCTGCGTTTATAAGGAGACACTTTATGTTTAAGAAGGTTGGTAAATTCCTTGCGGCTTTGGCAGCTATCCTGACGCTTGCGTATATTCTTGCGGTATACCCTCAAGTAGCACTAGTAGTAGTTGGCGCTTGTTACTTAGCGGCA‑‑GTGTGTGCt                                                                                                  >  1:156954/1‑301 (MQ=255)`  
                                                                                                                                                                                                              `cTTACGATGGCTGGGCGTGTCAAATTAGCTACATGGAGGAAACACCTGATGGCTCTTTGCGGCACCCATCGTTCGTAATGTTCCGTGGCACCGAGAACAACCCTCAAGAGAAAATGTAATCACACTGGCTCACCTTCGGGTGGGCCTTTCTGCGTTTATAAGGAGACACTTTATGTTTAAGAAGGTTGGTAAATTCCTTGCGGCTTTGGCAGCTATCCTGACGCTTGCGTATATTCTTGCGGTATACCCTCAAGTAGCACTAGTAGTAGTTGGCGCTTGTTACTTAGCGGCA‑‑GTGTGTGCt                                                                                                  <  1:287776/301‑1 (MQ=255)`  
                                                                                                                                                                                                              `cTTACCATGGCTCGGCGTGTGCTATTAGCTACCTGGACGAACCTACTGATGGCGCTTTGCGGCACCTATCCTTCGTAATGTCCCGTGACACCGAGGCCCACTGTAAAGCGACAATGTCTTCACCCTGGCTCGCCTTCGGTTAGTCTGTTCTTCGTTTCTAAGGTGCAACTTTATGTTTAAGAATGTTCGTAACTTCCTTTCGGCTTTGGCACCTATCCTGACGCTTGCGTATATTCTTGCGATATACCCTCAAGTATCACTAGTAGTAGTTGGCGCTTGTTACTTAGCGGCA‑‑GTGTGTGCt                                                                                                  <  1:272692/301‑1 (MQ=255)`  
                                                                                                                                                                                                                `tACGATGGCTGGGCGTGTCAAATTAGCTACATGGAGGAAACACCGGATGGCTCTTGGCGGCACCCATCGTTCGTAATGTTCCGTGGCACCGAGGACAACCCTCAAGAGAAAATGTAATCACACTGGCTCACCTTCGGGTGGGCCTGTCTGCGTTTATAAGGAGACACTTTATGTTTAAGAAGGTTGGTAAATTCCTTGCGGCTTTGGCAGCTATCCTGACGCTTGCGTATATTCTTGCGGTATACCCTCAAGTAGCACTAGTAGTAGTTGGCGCTTGTTACTTAGCGGCA‑‑GTGTGTGCTTg                                                                                                <  1:199851/301‑1 (MQ=255)`  
                                                                                                                                                                                                                      `ggCTGGGCGTGTCAAATTAGCTACATGGAGGAAACACCTGATGGCTCTTTGCGGCACCCATCGTTCGTAATGTTCCGTGGCACCGAGGACAACCCTCAAGAGAAAATGTAATCACACTGGCTCACCTTCGGGTGGGCCTTTCTGCGTTTATAAGGAGACACTTTATGTTTAAGAAGGTTGGTAAATTCATTGCGGCTTTGGCAGCTATCCTGACGCTTGCGTATATTCTTGCGGTATACCCTCAAGTAGCACTAGTAGTAGTTGGCGCTTGTTACTTAGCGGCA‑‑GTGTGTGCTTGCGtgtg                                                                                          >  1:301382/1‑301 (MQ=255)`  
                                                                                                                                                                                                                      `ggCTGGGCGTGTCAAATTAGCTACATGGAGGAAACACCTGATGGCTCTTTGCGGCACCCATCGTTCGTAATGTTCCGTGGCACCGAGGACAACCATCAAGAGAAAATGTAATCACACTGGCTCACCTTCTGGTGGGCCTTTCTGCGTTTATAAGGAGACACTTTATGTTTAAGAAGGTTGGTAAATTCCTTGCGGCTTTGGCAGCTATCCTGACGCTTGCGTATATTCTTGCGGTATACCCTCAAGTAGCACTAGTAGTAGTTGGCGCTTGTTACTTAGCGGCA‑‑GTGTGTGCTTGCGtgtg                                                                                          >  1:14028/1‑301 (MQ=255)`  
                                                                                                                                                                                                                         `ggggCGTGTCAAATTAGCTACATGGAGGAAACACCGGATGGCTCTTTGCGGCACCCATCGTTCGTAATGTTCCGTGGCACCGAGGACAACCCTCAAGAGAAAATGTAATCACACTGGCTCACCTTCGTGTGGGCCTTTCTGCGTTTATAAGGAGACACTTTATGTTTAAGAAGGTTGGTAAATTCCTTGCGGCTTTGGCAGCTATCCTGACGCTTGCGTATATTCTTGCGGTATACCCTCAAGTAGCACTAGTAGTAGTTGGCGCTTGTTACTTAGCGGCA‑‑GTGTGTGCTTGCGTGTGGAg                                                                                       <  1:329610/300‑1 (MQ=255)`  
                                                                                                                                                                                                                            `gCGTGTCAAATTAGCTACATGGAGGAAACACCTGATGGCTCTTTGCGGCACCCATCGTTCGTAATGTTACGTGGCACCGAGGACAACCCTCAAGAGAAAATGTAATCACACTGGCTCACCTTCGGGTGGGCCTTTCTGCGTTTATAAGGAGACACTTTATGTTTAAGAAGGTTGGTAAATTCCTTGCGGCTTTGGCAGCTATCCTGACGCTTGCGTATATTCTTGCGGTATACCCTCAAGTAGCACTAGTAGTAGTTGGCGCTTGTTACTTAGCGGCA‑‑GTGTGTGCTTGCGTGTGGAGtat                                                                                    <  1:28572/301‑1 (MQ=255)`  
                                                                                                                                                                                                                               `tgtCAAATTAGCTACATGGAGGAAACACCTGATGGCTCTTTGCGGCACCCATCGTTCGTAATGTTCCGTGGCACCGAGGACAACCCTCAAGAGAAAATGTAATCACACTGGCTCACCTTCGGGTGGGCCTTTCTGCGTTTATAAGGAGACACTTTATGTTTAAGAAGGTTGGTAAATTCCTTGCGGCTTTGGCAGCTATCCTGACGCTTGCGTATATTCTTGCGGTATACCCTCAAGTAGCACTAGTAGTAGTTGGCGCTTGTTACTTAGCGGCA‑‑GTGTGTGCTTGCGTGTGGAGTATAGt                                                                                 <  1:265044/301‑1 (MQ=255)`  
                                                                                                                                                                                                                               `tgtCAAATTAGCTACATGGAGGAAACACCTGATGGCTCTTTGCGGCACCCATCGTTCGTAATGTTCCGTGGCACCGAGGACAACCCTCAAGAGAAAATGTAATCACACTGGCTCACCTTCGGGTGGGCCTTTCTGCGTTTATAAGGAGACACTTTATGTTTAAGAAGGTTGGTAAATTCCTTGCGGCTTTGGCAGCTATCCTGACGCTTGCGTATATTCTTGCGGTATACCCTCAAGTAGCACTAGTAGTAGTTGGCGCTTGTTACTTAGCGGCA‑‑GTGTGTGCTTGCGTGTGGAGTATAGt                                                                                 <  1:232799/301‑1 (MQ=255)`  
                                                                                                                                                                                                                               `tgtCAAATTAGCTACATGGAGGAAACACCTGATGGCTCTTTGCGGCACCCATCGTTCGTAATGTTCCGTGGCACCGAGGACAACCCTCAAGAGAAAATGTAATCACACTGGCTCACCTTCGGGTGGGCCTTTCTGCGTTTATAAGGAGACACTTTATGTTTAAGAAGGTTGATAAATTCCTTGCGGCTTTGGCAGCTATCCTGACGCTTGCGTATATTCTTGCGGTATACCCTCAAGTAGCACTAGTAGTAGTTGGCGCTTGTTACTTAGCGGCA‑‑GTGTGTGCTTGCGTGTGGAGTATAGt                                                                                 >  1:160209/1‑301 (MQ=255)`  
                                                                                                                                                                                                                               `tgtCAAATTAGCTACATGGAGGAAACACCTGATGGCTCTTTGCGGCACCCATAGTTCGTAATGTTCCGTGGCACCGAGGACAACCCTCAAGAGAAAAGGTAATCACACTGGCTCACCTTCGGGTGGGCCTTTCTGCGTTTATAAGGAGACACTTTATGTTTAAGAAGGTTGGTAAATTCCTTGCGGCTTTGGCAGCTATCCTGACGCTTGCGTATATTCTTGCGGTATACCCTCAAGTAGCACTAGTAGTAGTTGGCGCTTGTTACTTAGCGGCA‑‑GTGTGTGCTTGCGTGTGGAGTATAGt                                                                                 <  1:222288/301‑1 (MQ=255)`  
                                                                                                                                                                                                                                 `tCAAATTAGCTACATGGAGGAAACACCTGATGGCTCTTTGCGGCACCCATCGTTCGTAATGTTCCGTGGCACCGAGGACAACCCTCAAGAGAAAATGTAATCACACTGGCTCACCTTCGGGTGGGCCTTTCTGCGTTTATAAGGAGACACTTTATGTTTAAGAAGGTTGGTAAATTCCTTGCGGCTTTGGCAGCTATCCTGACGCTTGCGTATATTCTTGCGGTATACCCTCAAGTAGCACTAGTAGTAGTTGGCGCTTGTTACTTAGCGGCA‑‑GTGTGTGCTTGCGTGTGGAGTATAGTTa                                                                               <  1:163470/301‑1 (MQ=255)`  
                                                                                                                                                                                                                                 `tCAAAGTAGCTACATGGAGGAAACACCTGATGGCTCTTTGCGGCACCCATCGTTCGTAATGTTCCGTGGCACCGAGGACAACCCTCAAGAGAAAATGTAATCACACTGGCTCACCTTCGGGTGGGCCTTTCTGCGTTTATAAGGAGACACTTTATGTTTAAGAAGGTTGGTAAATTCCTTGCGGCTTTGGCAGCTATCCTGACGCTTGCGTATATTCTTGCGGTATACCCTCAAGTAGGACTAGTAGTAGTTGGCGCTTGTTACTTAGCGGCA‑‑GTGTGTGCTTGCGTGTGGAGTATAGTTa                                                                               <  1:86551/301‑1 (MQ=255)`  
                                                                                                                                                                                                                                    `aaTTAGCTACATGGAGGAAACACCTGATGGCTCTTTGCGGCACCCATCGTTCGTAATGTTCCGTGGCACCGAGGACAACCCTCAAGAGAAAATGTAATCACACTGGCTCACCTTCGGGTGGGCCTTTCTGCGTTTATAAGGAGACACTTTATGTTTAAGAAGGTTGGTAAATTCCTTGCGGCTTTGGCAGCTATCCTGACGCTTGCGTATATTCTTGCGGTATACCCTCAAGTAGCACTAGTAGTAGTTGGCGCTTGTTACTTAGCGGCA‑‑GTGTGTGCTTGCGTTTGGAGTATAGTTAACt                                                                            >  1:182573/1‑301 (MQ=255)`  
                                                                                                                                                                                                                                     `aTTAGCTACATGGAGGAAACACCTGATGGCTCTTTGCGGCACCCATCGTTCGTAATGTTCCGTGGCACCGAGGACAACCCTCAAGAGAAAATGTAATCACACTGGCTCACCTTCGGGTGGGCCTTTCTGCGTTTATAAGGAGACACTTTATGTTTAAGAAGGTTGGTAAATTCCTTGCGGCTTTGGCAGCTATCCTGACGCTTGCGTATATTCTTGCGGTATACCCTCAAGTAGCACTAGTAGTAGTTGGCGCTTGTTACTTAGCGGCA‑‑GTGTGTGCTTGCGTGTGGAGTATAGTTAACTg                                                                           <  1:121652/301‑1 (MQ=255)`  
                                                                                                                                                                                                                                      `ttAGCTACATGGAGGAAACACCTGATGGCTCTTTGCGGCACCCATCGTGCGTAATGTTCCGTGGCACCTAGGACAACCCTCAAGAGAAAATGTAATCACACTGGCTCACCTTCGGGTGGGCCTTTCTGCGTTTATAAGGAGACACTTTATGTTTAAGAAGGTTGGTAAATTCCTTGCGGCTTTGGCAGCTATCCTGACGCTTGCGTATATTCTTGCGGTATACCCTCAAGTAGCACTAGTAGTAGTTGGCGCTTGTTACTTAGCGGCA‑‑GTGTGTGCTTGCGTGTGGAGTATAGTTAACTgg                                                                          <  1:81462/301‑1 (MQ=255)`  
                                                                                                                                                                                                                                      `ataagagacaggaggaAACCACTGATGGCTCTTTGCGGCACCCATCGTTCGTAATGTTCCGTGGCACCGAGGACAACCCTCAAGAGAAAATGTAATCACACTGGCTCACCTTCGGGTGGGCCTTTCTGCGTTTATAAGGAGACACTTTATGTTTAAGAAGGTTGGTAAATTCCTTGCGGCTTTGGCAGCTATCCTGACGCTTGCGTATATTCTTGCGGTATACCCTCAAGTAGCACTAGTAGTAGTTGGCGCTTGTTACTTAGCGGCA‑‑GTGTGTGCTTGCGTGTGGAGTATAGTTAACTgg                                                                          <  1:251071/291‑1 (MQ=255)`  
                                                                                                                                                                                                                                        `aGCTACATGGAGGAAACACCTGATGGCTCTTTGCGGCACCCATCGTTCGTAATGTTCCGTGGCACCGAGGACAACCCTCAAGAGAAAATGTATTCACACTGGCTCACCTTCGGATGGGCCTTTCTGCGTTTATAAGGAGACACTTTATGTTTAAGAAGGTTGGTAAATTCCTTGCGGCTTTGGCAGCTATCCTGACGCTTGCGTATATGCTTGCGGTATACCCTCAAGTAGCACTAGTAGTAGTTGGCGCTTGTTACTTAGCGGCA‑‑GTGTGTGCTTGCGTGTGGAGTATAGTTAACTGGTa                                                                        <  1:229957/301‑1 (MQ=255)`  
                                                                                                                                                                                                                                         `gcgaCATGGAGGAAACACCTGATGGCTCTTTGCGGCACCCATCGTTCGTAATGTTCCGTGGCACCGATGACAACCCTCAAGAGAAAATGTAATCACACTGGCTCACCTTCGGGTGGGCCTTTCTGCGTTTATAAGGAGACACTTGATGTTTAAGAAGGTTGGTAAATTCCTTGCGGCTTTGGCAGCTATCCTGACGCTTGCGTATATTCTGGCGGTATACCCTCAAGTAGCACTAGTAGTAGTTGGCGCTTGTTACTTAGCGGCA‑‑GTGTGTGCTTGCGTGTGGAGTATAGTTAACTGGTaa                                                                       <  1:36879/298‑1 (MQ=255)`  
                                                                                                                                                                                                                                         `gCTACATGGAGGAAACACCTGATGGCTCTTTGCGGCACCCATCGTTCGTAATGTTCCGTGGCACCGAGGACAACCCTCAAGAGAAAATGTAATCACACTGGCTCACCTTCGGGTGGGCCTTTCTGCGTTTATAAGGAGACACTTTATGTTTAAGAAGGTTGGTAAATTCCTTGCGGCTTTGGCAGCTATCCTGACGCTTGCGTATATTCTTGCGGTATACCCTCAAGTAGCACTAGTAGTAGTTGGCGCTTGTTACTTAGCGGCA‑‑GTGTGTGCTTGCGTGTGGGGTATAGTTAACTGGTaa                                                                       >  1:216573/1‑301 (MQ=255)`  
                                                                                                                                                                                                                                            `aCATGGAGGAAACACCTGATGGCTCTTTGCGGCACCCATCGTTCGTAATGTTCCGTGGCACCGAGGACAACCCTCAAGAGAGAATGTAATCACACTGGCTCACCTTCGGGTGGGCCTTTCTGCGTTTATAAGGAGGCACTTTATGTTTAAGAAGGTTGGTAAATTCCTTGCGGCTTTGGCAGCTATCCTGACGCTTGCGTATATTCTTGCGGTATACCCTCAAGTAGCACTAGTAGTAGTTGGCGCTTGTTACTTAGCGGCA‑‑GTGTGTGCTTGCGTGTGGCGTATAGTTAACTGGTAATAc                                                                    >  1:49900/1‑301 (MQ=255)`  
                                                                                                                                                                                                                                              `aTGGAGGAAACACCTGATGGCTCTTTGCGGCACCCATCGTTCGTAATGTTCCGTGGCACCGAGGACAACCCTCAAGAGAAAATGTAATCACACTGGCTCACCTTCGGGTGGGCCTTTCTGCGTTTATAAGGAGACACTTTATGTTTAAGAAGGTTGGTAAATTCCTTGCGGCTTTGGCAGCTATCCTGACGCTTGCGTATATTCTTGCGGTATACCCTCAAGTAGCACTAGTAGTAGTTGGCGCTTGTTACTTAGCGGCAGTGTGTGTGCTTGCGTGTTGAGTATAGTTAACTGGTAATAc                                                                    <  1:219390/301‑1 (MQ=255)`  
                                                                                                                                                                                                                                                    `aaaaCTCCTGATGACTCTTTGCGGCACCCATCGTTCGTAATGTTCCGTGGCACCGAGGACAACCCTCAAGAGAAAATGTAATCACACTGGCTCAGCTTCGTGTGGGCCTTTCTGCGTTTATAAGGAGACACTTTATGTTTAAGAAGGTTCGTAAATTCCTTGCGGCTTTGGCAGCTATCCTGACGCTTGCGTATATTCTTGCGGTATACCCTCAAGTAGCACTAGTAGTAGTTGGCGCTTGTTACTTAGCGGCA‑‑GTGTGTGCTTGCGTGTGGAGTATAGTTAACTGGTAATACGACTCACt                                                            <  1:311673/300‑1 (MQ=255)`  
                                                                                                                                                                                                                                                         `acCTGATGGCTCTTTGCGGCACCCATCTTTCGTAATGTTCCGTGGCACCGAGGACAACCCTCAAGAGAAAATGTAATCACACTGGCTCACCTTCGGGTGGGCCTTTCTGCGTTTATAAGGAGACACTTTATGTTTAAGAAGGTTGGTAAATTCCTTGCGGCTTTGGCAGCTATCCTGACGCTTGCGTATATTCTTGCGGTATACCCTCAAGTAGCACTAGTAGTAGTTGGCGCTTGTTACTTAGCGGCA‑‑GTGTGTGCTTGCGTGTGGAGTATAGTTAACTGGTAATACGACTCACTAAagg                                                       <  1:325516/301‑1 (MQ=255)`  
                                                                                                                                                                                                                                                         `acCTGATGGCTCTTTGCGGCACCCATCGTTCGTAATGTTACGTGGCACCGAGGACAACCCTCTAGAGAAAATGTAATCACACTGGCTCACCTTCGGGTGGGCCTTTCTGCGTTTATAAGGAGACACTTTATGTTTAAGAAGGTTGGTAAATTCCTTGCGGCTTTGGCAGCTATCCTGACGCTTGCGTATATTCTTGCGGTATACCCTCAAGTAGCACTAGTAGTAGTTGGCGCTTGTTACTTAGCGGCA‑‑GTGTGTGCTTGCGTGTGGAGTATAGTTAACTGGTAATACGACTCACTAAagg                                                       <  1:96493/301‑1 (MQ=255)`  
                                                                                                                                                                                                                                                          `ccTGATGGCTCTTTGCGGCACCCATCGTTCGTAATGTTCCGTGGCACCGAGGACAACCCTCAAGAGAAAATGTAATCACACTGGCTCACCTTCGGGTGGGCCTTTCTGCGTTTATAAGGAGACACTTTATGTTTAAGAAGGTTGGTAAATTCCTTGCGGCTTTTGCAGCTATCCTGACGCTTGCGTATATTCTTTCGGTATACCCTCAAGTAGCACTAGTAGTAGTTGGCGCTTGTTACTTAGCGGCA‑‑GTGTGTGCTTGCGTGTGGAGTATAGTTAAcctgtctcttatacacatctccga                                                      >  1:17316/1‑278 (MQ=255)`  
                                                                                                                                                                                                                                                                `ggCTCTTTGCGGCACCCATCGTTCGTAACGTTCCGTGGCACCTAGGACAACCCTCAAGAGAAAATGTAATCACACTGGCTCACCTTCGGGTTGGCCTTTCTGCGTTTATAAGGAGACACTTTATGTTTAAGAAGGTTGGTAAATTCCTTGCGGCTTTTGCAGCTATCCTGACGCTTGCGTATATTCTTGCGGTATACCCTCAAGTAGCACTAGTAGTAGTTGGCGCTTGTTACTTAGCGGCA‑‑GTGTGTGCTTGCGTGTGGAGTATAGTTAACTGGTAATACGACTCACTAAaggctgtctc                                                >  1:110009/1‑294 (MQ=255)`  
                                                                                                                                                                                                                                                                 `gCTCTTTGCGGCACCCATCGTTCGTAATGTTCCGTGGCACCGAGGACAACCCTCAAGAGAAAATGTAATCACACTGGCTCACCTTCGTGTGGGCCTATCTGCGTTTATAAGGAGACACTTTATGTTTAAGAAGGTTGGTAAATTCCTTGCGGCTTTGGCAGCTATCCTGACGCTTGCGTATATTCTTGCGGTATACCCTCAAGTAGCACTAGTAGTAGTTGGCGCTTGTTACTTAGCGGCA‑‑GTGTGTGCTTGCGTGTGGAGTATAGTTAACTGGTAATACGACTCACTAAAGGAGGT‑‑acac                                             >  1:71395/1‑301 (MQ=255)`  
                                                                                                                                                                                                                                                                      `ttGCGGCACCCATCGTTCGTAATGTTCCGTGGCACCGAGGACAACCCTCAAGAGAAAATGTAATCACACTGGCTCACCTTCGGGTGGGCCTTTCTGCGTTTATAAGTAGACACTTTATGTTTCTGAAGGTTGGTAAATTCCTTGCGGCTTTGGCAGCTATCCTGACGCTTGCGTATATTCTTGCGGTATACCCTCAAGTAGCAATAGTAGTAGTTGGCGCTTGTTACTTAACGGCA‑‑ATGTGTGCTTGCGTGTGTAGTATAGTTAACTGGTAATACGACTCACTAAAGGAGGT‑‑ACACACCat                                        >  1:143337/1‑301 (MQ=255)`  
                                                                                                                                                                                                                                                                          `gtgggctcggagatgtgtataagagacagaCCGAGGACAACCCTCAAGAGAAAATGTAATCACACTGGCTCACCTTCGGGTGGGCCTTTCTGCGTTTATAAGGAGACACTTTATGTTTAAGAAGGTTGGTAAATTCCTTGCGGCTTTGGCAGCTATCCTGACGCTTGCGTATATTCTTTCGGTATACCCTCAAGTAGCACTAGTAGTAGTTGGCGCTTGTTACTTAGCGGCA‑‑GTGTGTGCTTGCGTGTGGAGTATAGTTAACTGGTAATACGACTCACTAAAGGAGGT‑‑ACACACCatgatg                                    <  1:40383/272‑1 (MQ=255)`  
                                                                                                                                                                                                                                                                             `ctcggagatgtgtataagagacaggCACCGAGGACAACCCTCAAGAGAAAATGTAATCACACTGGCTCACCTTCGGGTGGGCCTTTCTGCGTTTATAAGGAGACACTTTATGTTTAAGAAGGTTGGTAAATTCCTTGCGGCTTTGGCAGCTATCCTGACGCTTGCGTATATTCTTGCGGTATACCCTCAAGTAGCACTAGTAGTAGTTGGCGCTTGTTACTTAGCGGCA‑‑GTGTGTGCTTGCGTGTGGAGTATAGTTAACTGGTAATACGACTCACTAAAGGAGGT‑‑ACACACCATGATGTAc                                 <  1:320039/278‑1 (MQ=255)`  
                                                                                                                                                                                                                                                                             `aCCCATCGTTCGTAATGTTCCGTGGCACCGAGGACAACCCTCAAGAGAAAATGTAATCACACTGGCTCACCTTCGGGTGGGCCTTTCTGCGTTTATAAGGAGACACTTTATGTTTAAGAAGGTTGGTAAATTACTTGCGGCTTTGGCAGCTATCCTGACGCTTGCGTATATTCTTTCGGTATACCCTCAAGTAGCACTAGTAGTAGTTGGCGCTTGTTACTTAGCGGCA‑‑GTGTGTGCTTGCGTGTGGAGTATAGTTAACTGGTAATACGCCTCACTAAAGGAGGT‑‑ACACACCatgactgtt                                 >  1:65302/1‑296 (MQ=255)`  
                                                                                                                                                                                                                                                                                     `ttcgtAATGTTCCGTGGCACCGAGGACAACCCTCAAGAGAAAATGTAATCACACTGGCTCACCTTCGGGTGGGCCTTTCTGCGTTTATAAGGAGACACTTTATGTTTAAGAAGGTTGGTAAATTCCTTGCGGCTTTGGCAGCTATCCTGACGCTTGCGTATATTCTTGCGGTATACCCTCAAGTAGCACTAGTAGTAGTTGGCGCTTGTTACTTAGCGGCA‑‑GTGTGTGCTTACGTGTGGAGTATAGTTAACTGGTAATACGACTCACTAAAGGAGGT‑‑ACAAACCATGATGTACTTAATGca                         >  1:254204/1‑300 (MQ=255)`  
                                                                                                                                                                                                                                                                                            `tGTTCCGTGGCACCGAGGACAACCCTCAATAGAAAATGTAATCACTCTGGCTCACCTTCGGGTGGGCCTTTCTGCGTTTATAAGGAGACACTTTATGTTTAAGAAGGTTGGTAAATTCCTTGCGGCTTTGGCAGCTATCCTGACGCTTGCGTATATTCTTGCGGTATACCCTCAAGTAGCACTAGTAGTAGTTGGCGCTTGTTACTTAGCGGCA‑‑GTGTGTGCTTGCGTGTGGAGTATAGTTAACTGGTAATACGACTCACTAAAGGAGGT‑‑ACACACCATGATGTACTTAATGCCATTACTc                  >  1:94300/1‑301 (MQ=255)`  
                                                                                                                                                                                                                                                                                               `tCCGTGGCACCGAGGACAACCCTCAAGAGAAAATGTAATCCCACTGGCTCACCTTCGGGTGGGCCTTTCTGCGTTTATAAGGAGACACTTTATGTTTAAGAATGTTGGTAAATTCCTTGCGGCTTTGGAAGCTATCCTGACGCTTGCGTATATTCTTGCGGTATACCCTCAAGTAGCACTAGTAGTAGTTGGCGCTTGTTACTTAGCGGCA‑‑GTGTGTGCTTGCGTGTGGAGTATAGTTAACTGGTAATACGACTCACTAAAGGAGGT‑‑ACACACCATGATGTACTTAATGCCATTACTCATc               <  1:227870/301‑1 (MQ=255)`  
                                                                                                                                                                                                                                                                                                     `gCACCGAGGACAACCCTCAAGAGAAAATGTAATCACACTGGCTCACCTTCGGGTGGGCCTTTCTGCGTTTATAAGGAGACACTTTATGTTTAAGAAGGTTGGTAAATTCCTTGCGGCTTTGGCAGCTATCCTGACGCTTGCGTATATTCTTGCGGTATACCCTCAAGTAGCACTAGTAGTAGTTGGCGCTTGTTACTTAGCGGCA‑‑GTGTGTGCTTGCGTGTGGAGTATAGTTAACTGGTAATACGACTCACTAAAGGAGGT‑‑ACACACCATGATGTACTTAATGCCATTACTCATCGTCAtt         >  1:185208/1‑301 (MQ=255)`  
                                                                                                                                                                                                                                                                                                       `aCCGAGGACAACCCTCAAGAGAAAATGTAATCACACTGGCTCACCTTCGGGTGGGCCTTTCTGCGTTTATAAGGAGACACTTTATGTTTAAGAAGGTTGGTAAATTCCTTGCGGCTTTGGCAGCTATCCTGACGCTTGCGTATATTCTTGCGGTATACCCTCAAGTAGCACTAGTAGTAGTTGGCGCTTGTTACTTAGCGGCA‑‑GTGTGTGCTTGCGTGTGGAGTATAGGTAACTGGGAATACGACTCACTAAAGGAGGT‑‑ACACACCATGATGTACTTAGTGCCGTTACTAATCGTCATTGt       >  1:130861/1‑301 (MQ=255)`  
                                                                                                                                                                                                                                                                                                       `aCCGAGGACAACCCTCAAGAGAAAATGTAATCACACTGGCTCACCTTCGGGTGGGCCTTTCTGCGTTTATAAGGAGACACTTTATGTTTAAGAAGGTTGGTAAATTCCTTGCGGCTTTGGCAGCTATACTGACGCTTGCGTATATTCTTGCGGTATACACTCAAGTAGCACTAGTAGTAGTTGGCGCTTGTTACTTAGCAGCA‑‑GTGTGTGCTTGCGTGTGGAGTATAGTTAACTGGTAATACGACTCACTAAAGGAGGT‑‑ACACACCATGATGTACTTAATGCCATTACTCATCGTCATTGt       <  1:57996/301‑1 (MQ=255)`  
                                                                                                                                                                                                                                                                                                          `gAGGACAACCCTCAAGAGAAAATGTAATCACACTGGCTCACCTGCGGGTGGGCCTTTCTGCGTTTATAAGGAGACACTTTATGTTTAGGAAGGTTGGTAAATTCCTTGCGGCTTTGGCAGCTATCCTGACGCTTGCGTATATTCTTGCGGTATACCCTCAAGTAGCACTAGTAGTAGTTGGCGCTTGTTACTTAGCGGCA‑‑GTGTGTGCTTGCGTGTGGAGTATAGTTAACTTGTAATACGACTCACTAAAGGAGGT‑‑ACACACCATGATGTACTTAATGCCATTACTCATCGTCATTGTAgg    <  1:197583/301‑1 (MQ=255)`  
                                                                                                                                                                                                                                                                                                            `ggACAACCCTCAAGAGAAAATGTAATCACACTGGCTCACCTTCGGGTGGGCCTTTCTGCGTTTATAAGGAGACACTTTATGTTTAAGAAGGTTGGTAAATTCCTTGCGGCTTTGGCAGCTATCCTGACGCTTGCGTATATTCTTGCGGTATACCCTCAAGTAGCACTAGTAGTAGTTGGCGCTTGTTACTTAGCGGCA‑‑GTGTGTGCTTGCGTGTGGAGTATAGTTAACTGGTAATACGACTCACTAAAGGAGGTACACACACCATGATGTACTTAATGCCATTACTCATCGTCATTGTAgg    <  1:293750/301‑1 (MQ=255)`  
                                                                                                                                                                                                                                                                                                            `ggACAACCCTCAAGAGAAAATGTAATCACACTGGCTCACCTTCGGGTGGGCCTTTCTGCGTTTATAAGGAGACACTTTATGTTTAAGAAGGTTGGTAAATTCCTTGCGGCTTTGGCAGCTATCCTGACGCTTGCGTATATTCTTGCGGTATACCCTCAAGTAGCACTAGTAGTAGTTGGCGCTTGTTACTTAGCGGCA‑‑GTGTGTGCTTGCGTGTGGAGTATAGTTAACTGGTAATACGACTCACTAAAGGAGGT‑‑ACACACCATGATGTACTTAATGCCATTACTCATCGTCATTGTAGGAt  <  1:74518/301‑1 (MQ=255)`  
                                                                                                                                                                                                                                                                                                            `ggACAACCCTCAAGAGAAAATGTAATCACACTGGCTCACCTTCGGGTGGGCCTTTCTGCGTTTATAAGGAGACACTTTATGTTTAAGAAGGTTGGTAAATTCCTTGCGGCTTTGGCAGCTATCCTGACGCTTGCGTATATTCTTGCGGTATACCCTCAAGTAGCACTAGTAGTAGTTGGCGCTTGTTACTTAGCGGCA‑‑GTGTGTGCTTGCGTGTGGAGTATAGTTAACTGGTAATACGACTCACTAAAGGAGGT‑‑ACACACCATGATGTACTTAATGCCATTACTCATCGTCATTGTAGGAt  >  1:170962/1‑301 (MQ=255)`  

                                                                                                                                                                                                                                                                                                            `|`                                                                                                                                                                                                                                                                                                                  
`GGTATGGGGTACAAAAGGTCTGGCTAATGAAGGTAAAGTGATTGGTTTTGAGGTGCTTCTTGAGAGTGGTCGTTTAGTTAACGCCACGAATATCTCTCGCGCCTTAATGGATGAGTTCACTGAGACAGTAAAAGAGGCCACCCTAAGTCAATGGGGATTCTTTAGCCCATACGGTATTGGCGACAACGATGCTTGTACTATTAACCCTTACGATGGCTGGGCGTGTCAAATTAGCTACATGGAGGAAACACCTGATGGCTCTTTGCGGCACCCATCGTTCGTAATGTTCCGTGGCACCGAGGACAACCCTCAAGAGAAAATGTAATCACACTGGCTCACCTTCGGGTGGGCCTTTCTGCGTTTATAAGGAGACACTTTATGTTTAAGAAGGTTGGTAAATTCCTTGCGGCTTTGGCAGCTATCCTGACGCTTGCGTATATTCTTGCGGTATACCCTCAAGTAGCACTAGTAGTAGTTGGCGCTTGTTACTTAGCGGCA‑‑GTGTGTGCTTGCGTGTGGAGTATAGTTAACTGGTAATACGACTCACTAAAGGAGGT‑‑ACACACCATGATGTACTTAATGCCATTACTCATCGTCATTGTAGGAT  >  NC_001604.1/7230‑7830`

  

Alignment Legend

Aligned base mismatch/match (shaded by quality score): `ATCG/ATCG` `< 3 ≤` `ATCG/ATCG` `< 11 ≤` `ATCG/ATCG` `< 24 ≤` `ATCG/ATCG` `< 36 ≤` `ATCG/ATCG` `< 38 ≤` `ATCG/ATCG`

Unaligned base: `atcg`    Masked matching base: `atcg`    Alignment gap: `‑`    Deleted base: `‑`

Reads not counted as support for junction

read\_name Not counted due to insufficient overlap past the breakpoint.

read\_name Not counted due to not crossing MOB target site duplication.
